# MiR-124 and the Underlying Therapeutic Promise of Neurodegenerative Disorders

**DOI:** 10.3389/fphar.2019.01555

**Published:** 2020-01-17

**Authors:** Dong Han, Xiaoyu Dong, Dongming Zheng, Jianfei Nao

**Affiliations:** Department of Neurology, Shengjing Hospital of China Medical University, Shenyang, China

**Keywords:** neurodegenerative disorders, microglia, inflammation, miR-124, therapy

## Abstract

Neurodegenerative disorders (NDDs) are a group of chronic progressive neurological diseases based on primary neurodegeneration. The common pathological characteristics of various NDDs are neuronal degeneration, deletion, glial cell proliferation, and hypertrophy at specific locations in the nervous system. Proliferation and hypertrophy of microglia are manifestations of inflammation. MicroRNAs (miRNAs) have emerged as pivotal regulators of glial cells. MiRNAs are small non-coding molecules that regulate gene expression. Altered expression of miRNAs has been associated with several NDD pathological processes, among which regulation of the inflammatory response is key and a research hotspot at present. At the same time, miRNAs are also biological markers for diagnosis and potential targets for treating NDDs. MiR-124 is highly conserved and enriched in the mammalian brain. Emerging studies have suggested that miR-124 is closely related to the pathogenesis of NDDs and may be an effective treatment strategy to reduce inflammation associated with NDDs. In this review, we describe a summary of general miRNA biology, implications in pathophysiology, the potential roles of miR-124 associated with inflammation, and the use of miRNA as a future biomarker and an application for NDD therapy.

## Introduction

Neurodegenerative disorders (NDDs) are a group of unexplained chronic progressive neurological diseases based on neuronal degeneration, including Parkinson’ disease (PD), Alzheimer’s disease (AD), amyotrophic lateral sclerosis (ALS) and Huntington’s disease (HD) (Gitler et al., 2017). The neuroinflammatory reactions in the pathogenesis of NDDs have received more and more attention, and it has become a research hotspot in related fields (Schain and Kreisl, 2017). The role of neuroinflammation in the progression of different neurological diseases varies according to its role in the severity and progression of the disease (Chen et al., 2016). Studies have confirmed that the occurrence and development of many NDDs are closely related to neuroinflammation, among which activation of microglia is considered to be the key factor contributing to the upsurge (Tang and Le, 2016). Microglia are the resident macrophages of the central nervous system (CNS), accounting for about 10% of the total number of adult brain cells. Microglia play the important role of immune defense guardian and are responsible for protecting the brain from damage and invasion by pathogens. When neurons are damaged, microglia activate and undergo morphological changes, releasing a series of inflammatory cytokines to alleviate neuronal damage. However, microglia are also a “double-edged sword” (Li and Barres, 2018). Chronic inflammatory stimuli persist in NDDs, and activation of microglia results in the release of inflammatory factors, resulting in further neuronal damage.

## Neurodegenerative Disorders and Inflammation

**Figure 1:**
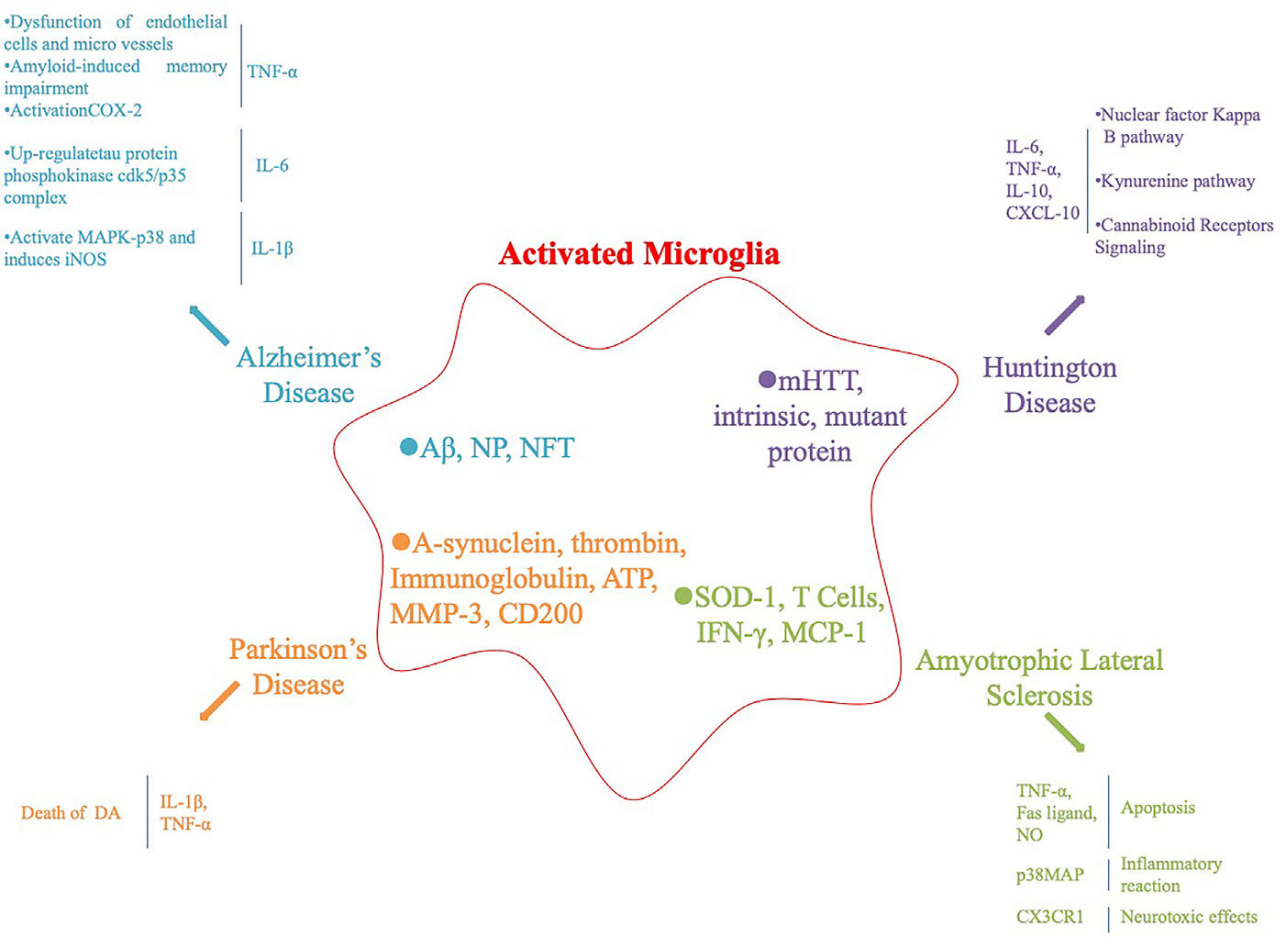
Activation of microglia and subsequent inflammatory factors released in neurodegenerative diseases and their animal models.

Microglia are an important component of the nervous system, including central and peripheral glial cells. Central glial cells mainly include astrocytes, oligodendrocytes microglia, and ependymal cells. Microglia are small in size and the main cells involved in the nervous system inflammatory response. They have the function of phagocytosis and clearing of cell debris and are the “macrophages” of the brain. Microglia are divided into resting and activated cells. Resting microglia are small and do not participate in the inflammatory reaction, whereas activated microglia are larger in volume, have a phagocytic function and participate in the inflammatory reaction. Under the influence of various factors, microglia secrete various cytokines, such as interferon-γ (IFN-γ), interleukin-β (IL-β), and tumor necrosis factor-α (TNF-α), which are involved in the inflammatory response (Colonna and Butovsky, 2017; Salter and Stevens, 2017), **Figure 1**.

AD has been associated with chronic neuroinflammation and microglia activation (McKenzie et al., 2017). Under the condition of AD pathology, dangerous-associated molecular patterns, amyloid-β peptide (Aβ), neurotrophic plaques (NPs), neurofibrillary tangles activate pattern recognition receptors (PRRs) and related complements on microglia and neuron surfaces cause a chronic inflammatory response, leading to neuronal damage (Salminen et al., 2009). PRRs include Toll-like receptors, formyl peptide receptors, NOD-like receptors, receptors for advanced glycation end products, and the α7-nicotinic acetylcholine receptor (Jiang et al., 2010). Activated microglia produce potential neurotoxic inflammatory factors, and the main AD-related inflammatory factors are TNF-α, IL-1, and IL-6. TNF-α is an inflammatory signaling factor that triggers a downstream cascade and regulates synaptic connections between neurons through corresponding receptors. Elevated levels of TNF-α in cerebrospinal fluid lead to synaptic dysfunction in patients with AD. Similarly, TNF-α also leads to dysfunction of endothelial cells and microvessels, amyloid production, and amyloid-induced memory impairment in patients with AD. Aβ activates the TNF-α-dependent intracellular signal transduction pathway, leading to upregulation and activation of cyclooxygenase-2 (COX-2) expression, which ultimately leads to neuronal synaptic loss and cognitive decline (Tobinick, 2009; Medeiros et al., 2010). IL-6 exists during the early stage of NP formation and increases significantly in patients with AD. IL-1 and IL-6 co-activate to upregulate expression of the tau protein phosphokinase cdk5/p35 complex. IL-1β activates mitogen activated protein kinase (MAPK)-p38 and induces inducible nitric oxide synthase (iNOS), leading to neuronal damage and death (Rojo et al., 2008). Although microglia protect the CNS by phagocytosis of Aβ and synaptic strippers, continuously activated microglia produce a variety of inflammatory factors and neurotoxins, leading to neuronal damage, which has a negative impact on the occurrence of AD.

PD is a common neurodegenerative disease in the elderly. Pathological changes include degenerative loss of dopaminergic neurons in the substantia nigra and formation of eosinophilic inclusion bodies in neurons. Malu et al. reported numerous activated microglia and cytokines in the substantia nigra compacta of PD patients during autopsy, further suggesting that activation of microglia is related to the degeneration of dopaminergic (DA) neurons (Tansey and Goldberg, 2010). Gerhard et al. (2006) reported obvious inflammatory reactions in the pons, basal ganglia, striatum, and frontotemporal lobe of patients with PD, suggesting that microglia in specific parts of the brain of patients with PD are activated during the early stage of disease. A growing body of research has confirmed that a variety of pathological changes in patients with PD activate microglia, including the main component of Lewy bodies (α-synuclein), thrombin and immunoglobulin activated protease-activated receptor-1 and Fc receptors; ATP released by injured neurons binds to the purine receptor; matrix metalloproteinase 3 (MMP-3); neurons in the inflammatory response, CD200 surface expression is downregulated, and inhibiting CD200 can activate microglia and induce microglia to release neurotoxic inflammatory factors (Kim et al., 2007; Lyons et al., 2007; Gu et al., 2010; Fan et al., 2015). Inflammatory factors are highly expressed in the substantia nigra striatum of 1-methyl-4-phenyl-1,2,3,6-tetrahydropyridine (MPTP) induced PD mice and in autopsy specimens of patients. IL-1β directly induces the death of DA neurons. Gayle et al. blocked IL-1β and TNF-α with neutralizing antibodies to show that nearly 50% of the death of DA neurons induced by lipopolysaccharide (LPS) were caused by these two factors. Therefore, inflammatory factors secreted by activated glial cells are involved in the degeneration of DA neurons. In addition, activated microglia accelerate the progress of PD by inducing oxidative stress and cytokine toxicity through inflammatory factors (Zhang et al., 2017). The microglia reaction plays a much more destructive than beneficial role in PD. Although the microglia reaction is not necessarily the initial step causing degeneration of DA neurons in the substantia nigra parotid, the microglia reaction is likely to involve and accelerate degeneration of DA neurons. The treatment to inhibit gliosis and iNOS-induced neurodegeneration represents one of the most promising therapeutic strategies for delaying and even preventing PD progression. Therefore, studying the relationship between microglia activation and PD will help to elucidate the etiology and pathogenesis of PD.

HD is an autosomal dominant inheritance disease affecting the striatum and cerebral cortex with a complete penetration rate of 50% in the offspring of affected individuals. HD is caused by *Huntingtin* mutation on chromosome 4 short arm 4p16.3. The gene product is CAG trinucleotide repeat amplification to produce Huntingtin protein. There are 11 to 34 CAG repeats in normal people, and HD is more than 40 (Ross and Tabrizi, 2011). HD is no exception, as there is increasing evidence that activated microglia can be detected in the brains of HD carriers and the post-mortem HD patients. In particular, elevated inflammatory cytokines are detected in the CNS and plasma of HD patients (Politis et al., 2015; Yang et al., 2017). Neuronal expression of mutant HTT and intrinsic mutant protein may be responsible for activating microglia. Activated microglia are present in a continuum of two functional states of polarization (M1 and M2 phenotypes) in which they extend damage to neighboring cells and then release different inflammatory factors (Yang et al., 2017). These inflammatory factors include pro-inflammatory cytokines (TNF-α and IL-1 β), chemokines (CCL2), MMP-9, IL-10, TGF-β, vascular endothelial growth factor, and insulin-like growth factor-1, which further activate the microglia activation signaling pathway in HD (Chhor et al., 2013; Cianciulli et al., 2015; Franco and Fernandez-Suarez, 2015; Orihuela et al., 2016).

ALS is a chronic and lethal NDD involving mainly motor neurons in the cerebral cortex, brainstem, and spinal cord. Neuroinflammation is observed in superoxide dismutase 1 (SOD-1)-transgenic mice and in patients with sporadic and familial ALS. Activation of microglia occurs before the loss of motor neurons (Golko-Perez et al., 2016). David et al. demonstrated that microglia accumulate in the spinal cord and peripheral nerves during the early stage of ALS and reported that activation of microglia is earlier than the clinical manifestation of ALS, indicating that microglia activate during the early stage of ALS leading to progression of the disease (Graber et al., 2010). SOD-1 activates microglia directly. In addition, T cells activate microglia by directly contacting or releasing proinflammatory factors (IFN-γ). The chemokine monocyte chemoattractant protein-1 (MCP-1) in cerebrospinal fluid chemotactically aggregates a large number of microglia into motor neurons (Boillee et al., 2006; Frank-Cannon et al., 2009). Activated microglia lead to neuronal death through the following pathways: the TNF-α-mediated apoptotic mechanism and Fas ligand or the nitric oxide-induced apoptotic pathway (Sargsyan et al., 2005). In addition, p38MAPK activation further increases the synthesis of inflammatory factors and promotes the inflammatory response; NF-κB activation leads to increased transcription of a series of inflammatory-related genes, including IL-2, IL-6, IL-8, IL-12p4, COX-2, iNOS, MMP-9, and MCP-1. Increased expression of the fractalkine receptor (CX3CR1) produces neurotoxic effects (Sargsyan et al., 2005; D’Ambrosi et al., 2009; Keizman et al., 2009). Inflammatory and cytotoxic factors released by activated microglia can induce or enhance progressive and selective motor neuron degeneration. However, the ALS inflammatory response involving microglia is complex. Although the specific mechanisms are still unclear, the interventions designed for microglia have a very attractive prospect, which may provide an effective treatment strategy for ALS.

## MiR Function

MiRs are small single-stranded RNAs of about 21–23 bases. They are produced by Dicer enzyme processing of about 70–90 single-stranded RNA precursors with a hairpin structure (Khandelwal et al., 2019). The first confirmed miRs were lin-4 and let-7 found in nematodes. Subsequently, several research groups began work on humans, fruit flies, and plants, and hundreds of miRs have been identified from a variety of species (He and Wang, 2012). MiRs are involved in a series of biological processes such as cell proliferation and apoptosis, growth and development, metabolic activation, and DNA repair *in vivo*, which are closely related to the occurrence and development of various diseases, especially tumors. MiRs regulate the target gene mRNA mainly by cutting off the target gene’s RNA molecule, inhibiting target gene translation, and inhibiting combining (Aalaei-Andabili and Rezaei, 2016). Specific expression or enrichment of miRs in mammalian brain tissues have been detected, such as miR-9, miR-124, and miR-132 (Lagos-Quintana et al., 2002; Lopez-Ramirez and Nicoli, 2014). MiR-124, for example, accounts for 25–48% of total mRNA in the adult brain and is highly expressed in all brain regions except the pituitary. MiR-124 is also considered to be a neuron-specific miR because it is mainly expressed in neuronal cells (Lagos-Quintana et al., 2002; Mishima et al., 2007). MiR-124 is temporarily upregulated during neurodevelopment and adult neurogenesis (Smirnova et al., 2005; Cheng et al., 2009). This miR also plays a key role regulating synaptic plasticity and memory signaling molecules (Fischbach and Carew, 2009). MiR-124 is also highly expressed in microglia, where it takes part in the regulation of microglia quiescence (Ponomarev et al., 2011; Saraiva et al., 2017).

## MiR-124 in Neurodegenerative Disorders

MiR-124 is one of the most abundant and best studied miRNAs that functions within the nervous and immune systems (Soreq and Wolf, 2011). MiR-124 has well-established functions during neuronal development and neurodegeneration. The level of miR-124 remains high in post-mitotic neurons, suggesting that it takes part in maintenance of the differentiated state of neurons (Ponomarev et al., 2011; Soreq and Wolf, 2011). MiR-124 also contributes to pathological conditions, while influencing microglia to adopt a surveillance state or anti-inflammatory phenotype (Coolen et al., 2013; Akerblom and Jakobsson, 2014) (**Table 1**). Previous studies have claimed that CNS inflammation is more prominent during the early stage of AD and microglia become less responsive to stimulation with age (Wojtera et al., 2012; Njie et al., 2012; Caldeira et al., 2014) provided the inflammation-miRNA aging characteristics of primary cultured microglia and confirmed the low expression of miR-124 in age-related neurodegenerative diseases at the cellular level through *in vitro* microglial studies at different time points. The mechanism of miR-124 involvement in AD is mainly to interfere with clearance of the amyloid precursor protein (APP). Fang et al. (2012) used PC 12 cellular AD models and suggested that miR-124 dysregulation is related to AD pathology by targeting BACE1 (𝛽-site APP cleaving enzyme 1), which is a key cleaver of the APP that plays a pivotal role in 𝛽-amyloid production. Those researchers demonstrated that there was a negative regulatory relationship between miR-124 and BACE1 expression, and that miR-124 could be a promising therapeutic target in patients with AD. In brain samples of patients with AD and mouse model, Smith et al. (2011) found that the expression of miR-124 was downregulated and concluded that miR-124 participates in post-transcriptional regulation of APP expression. As abnormal neuronal splicing of APP affects the production of 𝛽-amyloid peptide, their results contribute to understanding the significance of miRNAs in the pathogenesis of AD. Synaptic pathological changes usually occur during the early period of AD. Other mechanisms by which miR-124 is involved in the pathogenesis of AD have also been reported. Wang et al. (2018) revealed in *in vitro* cell studies of AD patients and Tg2576 mice (a recognized AD mouse model) that tyrosine-protein phosphatase non-receptor type 1 (PTPN1) is a direct target of miR-124, and that dysregulation of miR-124 leads to dysfunctional synaptic transmission and plasticity. Affecting the miR-124/PTPN1 pathway could restore synaptic failure and memory deficits. Villela et al. (2016) revealed that miR-124 is one of the most significantly dysregulated miRs in temporal cortex samples from patients with AD and predicted that dysregulation of miR-124 expression in the human brain may contribute to the pathogenic changes of AD (Villela et al., 2016). Aberrant activation of cyclin-dependent kinase-5 (CDK5) is mediated by calpain (CAPN)-induced cleavage of p35 into p25. Zhou et al. (2019) confirmed that miR-124 is a CAPN1-targeting miRNA, which functionally inhibits CAPN1 protein translation of HCN-2 cells. Transfection with miR-124-3p *in vitro* reduces levels of the CAPN1 protein, thereby attenuating CDK5-mediated hyperphosphorylation of APP and the tau protein. MiR-124 has been suggested to be associated with microvascular dysfunction and subsequent cognitive impairment. Downregulated expression of miR-124 results in Aβ deposition, a decline in microvascular density, and reduced angiogenesis (Li et al., 2019). Regulating miR-124 expression may prevent the onset and progression of AD through various pathways, and provide new ideas for treating AD.

**Table 1:** Overview of the miRNA-124 expression in the models of neurodegenerative disorders.

Neurodegenerative disorders	Model	MiRNA-124 expression	Molecules regulation	Possible biological function/pathological process	References
Alzheimer’s disease	PC 12 cell lines and SD rat	Downregulated	BACE1	Cleavage of amyloid precursor protein	Fang et al., 2012
	Human and Neuro2a cells	Downregulated	PTBP1	Amyloid precursor protein alternative splicing in neurons	Smith et al., 2011
	Tg2576 mice	Upregulated	PTPN1	Synaptic transmission and plasticity	Wang et al., 2016
	HCN-2	Downregulated	CAPN1	Post-transcriptional control	Zhou et al., 2018
Parkinson’ disease	Mouse brain tissue, BV2 cell	Downregulated	MEKK3 and p-p65	MEKK3/NF-κB signaling pathways	Yao et al., 2018
	Mouse brain tissue, SH-SY5Y	Downregulated	p62 and p38	Autophagy, microglial activation	Yao et al., 2019
	PC12 cells and SH-SY5Y cells	Downregulated	ANXA5	ANXA5/ERK pathway	Dong et al., 2018
	Mice model	Downregulated	CDK5	Calpain 1/cdk5 pathway	Kanagaraj et al., 2014
Huntington’s disease	Bioinformatic analysis	Downregulated	REST	Post-transcriptional control	Johnson and Buckley, 2009
Amyotrophic lateral sclerosis	NSC-34 cells	Upregulated	NF-kB activation	NF-kB activation and inflammatory factors release	Pinto et al., 2017
	SOD1-G93A mice	Upregulated	STAT3	Transcriptional control	Marcuzzo et al., 2014

A variety of pathogenic factors are involved in the onset and progression of PD, including inflammation, particularly the microglia-mediated chronic inflammatory reaction in the brain, and miR-124 is also involved. Gong et al. reported that miR-124 was downregulated in MPTP-induced neurons and that suppressing miR-124 significantly increased cell apoptosis and autophagy-associated protein expression of DA neurons in patients with PD (Gong et al., 2016). It has been suggested that miR-124 is significantly downregulated in the MPTP-induced PD mouse model and that it inhibits neuroinflammation during the development of PD (Ridolfi and Abdel-Haq, 2017; Yao et al., 2018). Yao et al. (2019) reported that miR-124 inhibits inflammation during the development of PD by targeting p38, p62, and autophagy, suggesting that miR-124 may be a promising treatment target for regulating the inflammatory response in patients with PD. Dong et al. (2018) evaluated the mechanism and effects of miR-124 on SH-SY5Y cells and 6-hydroxydopamine (6-OHDA)-induced neurotoxicity in PC12 and reported that the miR-124-3p level was downregulated. They also revealed that annexinA5 (ANXA5) is a direct target gene of miR-124-3p, which may attenuate 6-OHDA-induced neuron injury by regulating the ANXA5/ERK pathway. MiR-124 is also involved in increasing CDK5 expression mediated by calpain 1/p25 in the MPTP-mouse model of PD. Supplementing with miR-124 *in vitro* increases cell viability and attenuates protein expression of the calpain 1/cdk5 pathway (Kanagaraj et al., 2014). Yao et al. (2018) also suggested that overexpression of miR-124 in microglia could attenuate the MEKK3/NF-κB inflammatory pathway and limit the pathogenesis of PD. Previous studies have indicated that the mechanism of miR-124 in the pathogenesis of PD is relatively clear. That is to say, downregulated miR-124 promotes the pathogenesis of PD through a variety of mechanisms, and over-expression of miR-124 may play a therapeutic role in PD by preventing the inflammatory response.

There are a few studies on the relationship between miR-124 and HD. It was reported that miR-124 was downregulated in the cortex and hippocampus of HD transgenic mice (Johnson et al., 2008; Lee et al., 2017). The role of RE1-silencing transcription factor (REST) in the pathogenesis of HD has been fully studied. However, miR-124 is encoded by multiple genes each targeted by REST (Johnson and Buckley, 2009).

MiR-124 is related to different phenotypes and functions of microglia, which may be a different cause of neuroinflammation in patients with ALS (Brites and Vaz, 2014; Cunha et al., 2018). Significantly upregulated miR-124 was revealed by Pinto et al. (2017) in NSC-34 cells (a MN-like cell line) expressing SOD1G93A. Elevated miR-124 levels are thought to have a calming effect on microglia, as miR-124 is expressed by stressed neurons associated with neurodegeneration (Ponomarev et al., 2011). In addition, the elevated levels seem to be negatively correlated with the STAT3 transcription factor, a key factor for neural differentiation and cell survival (Marcuzzo et al., 2014). In a study of neural stem cells from ALS transgenic mice, Zhou et al. (2018) showed that miR-124 was downregulated in the brainstem and spinal cord, and that miR-124 may participate in astrocyte differentiation of ALS transgenic mice by targeting Sox2 and Sox9. Many studies have reported that the expression level of miR-124 is inconsistent in ALS models, which may be related to the samples selected by different studies, and more studies in the future may draw more clear conclusions. However, it is certain that miR-124 is involved in ALS through the inflammatory reaction. It will be possible to treat ALS with miR-124 as the target after further study on the pathological mechanism of ALS.

## MiR-124-Based Therapeutic Strategies and MiR Delivery

Although we are far from understanding the complete NDD picture, we now know that there is a clear correlation between the expression of miRNAs and neurodegeneration (Sadlon et al., 2019). Therapeutic strategies based on miRNAs can be direct, such as regulating target protein expression by increasing or decreasing specific levels of miRNAs, or indirect, by activating or enhancing endogenous repair mechanisms in the brain by introducing miRNAs. However, as an important potential target of NDD treatment, miRNAs still have the following shortcomings: (1) they are highly unstable and easily degraded by nucleases in the plasma; (2) because of their negative charge, miRNAs are difficult to pass through the cell membrane formed by a lipid bimolecular layer and cannot be released in the cell, resulting in low bioavailability; (3) systemic administration requires miRNAs to be targeted. Therefore, selecting a safe and efficient delivery vector is the key to the success of miRNA molecular NDD gene therapy (Cao et al., 2016; Brown et al., 2018; Szelenberger et al., 2019; Zetterberg and Burnham, 2019).

Viral and non-viral vectors have been developed to improve the delivery efficiency of miRNAs. The delivery system based on a virus as the carrier is highly efficient and can deliver miRNAs to target cells effectively. For example, Zhou et al. (2019) administered an intracranial injection of adeno-associated virus (AAV) expressing microRNA-124-3p into APP/PS1-AD mice, showing that A𝛽 deposition was significantly reduced and AD-mouse behavior and cognitive function were significantly improved. A similar approach has been used to treat PD. Yao et al. (2019) reported that exogenous delivery of miR-124 suppresses the expression of p38 and p62 and attenuates microglia activation in the substantia nigra pars compacta of MPTP-treated mice. However, the disadvantages of viral vectors (such as the immunogenicity/inflammatory response and low loading capacity) limit their application in gene delivery and make it difficult to achieve large-scale manufacturing and quality control (Itaka and Kataoka, 2009; Ma et al., 2018; Pfister et al., 2018). Compared with viral vectors, non-viral vector delivery systems (liposomes, polymer-based systems, and inorganic nanoparticles) are diverse and relatively safe and can effectively avoid the problems faced by viral vectors through reasonable design and appropriate modification. Therefore, non-viral vector delivery systems are widely used in clinical research (Yin et al., 2014; Labatut and Mattheolabakis, 2018). Liposomes have advantages of simple preparation, safety, and non-toxicity. Cationic liposomes or neutral liposomes have been widely studied as miRNA delivery carriers (Hobel and Aigner, 2013). Nanoparticles used for *in vivo* delivery of liposomes include cationic liposomes, neutral liposomes, and polyethylene glycol-modified liposomes. Liposome nanoparticles stabilize nucleic acids, resist enzyme degradation, promote cell uptake, and prolong the cycle half-life of miRNA (Hugon and Paquet, 2008; Hsu et al., 2013; Abu Lila and Ishida, 2017). Negative hydrophilic miRNA interacts with the positive liposome to form a complex system through a positive and negative charge interaction, and interact with the negative cell membrane to deliver miRNA. The efficiency of the combined miRNA uptake by the cell is also improved (Obeid et al., 2017; Tapeinos et al., 2017). Sasidharan et al. (2017) developed a novel liposome-based method for delivering anti-miR-124 efficiently into live planarians to demonstrate the roles of the miR-124 family of miRs in regulating the regeneration of a functional brain. Poly lactic-co-glycolic acid (PLGA) is a Food and Drug Administration approved biocompatible polymer, which can be hydrolyzed into nontoxic lactic acid and glycolic acid monomers and metabolized by the human body without any side effects (Devulapally and Paulmurugan, 2014). PLGA polymer nanoparticles capture bioactive molecules, escape from cytolysin to the cytoplasm, induce the sustained release of transported substances in cells, and prolong efficacy. Furthermore, introducing cationic polyethyleneimine with a positive charge into the PLGA system solves the problems of low molecular weight and low over efficiency of PLGA. At present, the PLGA carrier has been widely used to deliver various therapeutic genes (Devulapally and Paulmurugan, 2014; Cai et al., 2017; Malik and Bahal, 2019). PLGA nanoparticles have been used to efficiently deliver nicotine to the brain to achieve neuroprotective effects in PD caused by reactive oxygen species (Fornaguera et al., 2015). Yu et al. successfully delivered ketoprofen and miR-124 co-loaded PLGA microspheres to adjuvant-induced arthritic rats and demonstrated therapeutic efficiency (Yu et al., 2018). Gold nanoparticles (AuNPs) are of great interest as non-viral gene delivery vectors due to their ease of control in size and shape, and their biocompatibility and low cytotoxicity (Torrisi, 2015; Yoon et al., 2017). AuNPs maintain high stability in salt solution and specifically bind to complementary nucleic acids. In addition, magnetic nanoparticles can be used to deliver genetic material to a target site by means of an external magnetic field, thereby overcoming the disadvantage of poor transfection efficiency of the viral vector. This method reduces side effects, increases selectivity, and reduces the cost and dosage of non-viral vectors. Magnetic nanoparticles are primarily used to effectively treat cancer (Wang et al., 2016; Shabestari Khiabani et al., 2017). Saraiva et al. (2016) successfully constructed miRNA-124-loaded polymeric nanoparticles and injected them into a 6-OHDA-challenged mouse model of PD ventricles. The results showed that miRNA-124 NPs enhanced the migration of new neurons into the 6-OHDA lesioned striatum, culminating in improved motor function. Moreover, an exosome-based delivery method has also been successfully applied in HD research. Exo-124 (exosomes exhibit a high level of miR-124 expression) was injected into the striatum of R6/2 transgenic HD mice, and Lee et al. (2017) revealed that expression of the REST target gene decreased, which participates in multiple links in the pathogenesis of HD. While no miRNA therapeutics have entered clinical trials to treat NDDs, further advances in delivering miRNA therapeutics to the desired site of action may contribute to clinical development.

## Conclusions

Many processes are involved in the pathogenesis of NDDs, including transcriptional dysregulation, mitochondrial dysfunction, and synaptic dysfunction. Activation of microglia and inflammation are important pathogenic processes in NDDs. MiR-124 is closely related to the pathogenesis of NDDs and is downregulated in response to pro-inflammatory stimuli. Over-expressing miR-124 may be a viable strategy to reduce the inflammatory response associated with NDDs. However, more studies are required to investigate the specific effects of miR-124 in NDDs. Improved miR delivery methods in clinical trials may lead to the development of microglia-specific miR therapies for NDDs.

## Author Contributions

This manuscript was primarily written by XD. Figures were produced by DH and JN. All authors read and approved the final manuscript.

## Funding

This work was supported by Liaoning Provincial Key R&D Program Guidance Plan (grant no. 2018225091).

## Conflict of Interest

The authors declare that the research was conducted in the absence of any commercial or financial relationships that could be construed as a potential conflict of interest.
